# A Combined Random Forests and Active Contour Model Approach for Fully Automatic Segmentation of the Left Atrium in Volumetric MRI

**DOI:** 10.1155/2017/8381094

**Published:** 2017-02-19

**Authors:** Chao Ma, Gongning Luo, Kuanquan Wang

**Affiliations:** Biocomputing Research Center, School of Computer Science and Technology, Harbin Institute of Technology, Harbin 150001, China

## Abstract

Segmentation of the left atrium (LA) from cardiac magnetic resonance imaging (MRI) datasets is of great importance for image guided atrial fibrillation ablation, LA fibrosis quantification, and cardiac biophysical modelling. However, automated LA segmentation from cardiac MRI is challenging due to limited image resolution, considerable variability in anatomical structures across subjects, and dynamic motion of the heart. In this work, we propose a combined random forests (RFs) and active contour model (ACM) approach for fully automatic segmentation of the LA from cardiac volumetric MRI. Specifically, we employ the RFs within an autocontext scheme to effectively integrate contextual and appearance information from multisource images together for LA shape inferring. The inferred shape is then incorporated into a volume-scalable ACM for further improving the segmentation accuracy. We validated the proposed method on the cardiac volumetric MRI datasets from the STACOM 2013 and HVSMR 2016 databases and showed that it outperforms other latest automated LA segmentation methods. Validation metrics, average Dice coefficient (DC) and average surface-to-surface distance (S2S), were computed as 0.9227 ± 0.0598 and 1.14 ± 1.205 mm, versus those of 0.6222–0.878 and 1.34–8.72 mm, obtained by other methods, respectively.

## 1. Introduction

Atrial fibrillation (AF) is the most common cardiac electrical disorder and a major cause of stroke [1]. During the past decade, ablation of AF has become a commonly performed therapy procedure in many major hospitals throughout the world [2]. Accurate segmentation of the LA anatomy from MR images is of great importance for ablation guidance during the therapy procedure, automatically quantifying the LA fibrosis which is highly associated with postablation AF recurrence [3] and creating cardiac biophysical models [4, 5].

However, developing automated LA segmentation techniques is technically challenging due to several reasons. First, the myocardial walls of the LA are not uniform in thickness, and some area of the walls can be very thin, 2.3 ± 0.9 mm [6], which make it challenging to image in cardiac MRI at even the best resolutions available. Although the LA areas can be defined through intensity gradients between the blood pool and surrounding tissues, the adjacent anatomical structures, such as other cardiac chambers, the descending aorta, and the coronary sinus, present signal intensities similar to that of the blood pool, and even manual segmentations by expert raters may show significant variations. Furthermore, the LA structures vary considerably across subjects in terms of the topological variants of the pulmonary vein (PV) and the shape and size of the LA appendage (LAA) [6], prohibiting the use of strong statistical constraints. Finally, the boundary between the LA and the left ventricle is difficult to define due to the different opening positions of the mitral valve (MV) leaflets [7].

In the past several years, a number of techniques were applied to the heart chambers segmentation, in meeting the variety of needs of clinical diagnosing and therapy, including active contour models, graph cuts, and machine learning as well as knowledge-based approaches, such as statistical shape models or atlas-based methods. Compared to the literature of cardiac ventricles segmentation, the one of LA segmentation is much less abundant. In the LA segmentation field, random forests and active contour models and their variants are indeed particularly popular.

Random forests (RFs) [8] machine learning framework has recently enjoyed the increased attentions in the medical image segmentation [9–11]. The RFs are inherently suited for handling a high number of multiclass data with high data dimension and have proven to be accurate and robust for many cardiac tissue segmentation tasks [12]. For example, Margeta et al. [13] proposed to automatically separate the LA from other blood pools in 3D cardiac MRI by using context-rich features within a decision forests scheme. Schneider et al. [14] proposed a framework for joint 3D vessel segmentation and centerline extraction by using multivariate Hough voting and oblique RFs, with local image features extracted by steerable filters. Mahapatra [15] used RF to learn discriminative image features and quantify their importance. The learned feature selection strategy then guided the graph cut to achieve the final segmentation. Although these methods have achieved promising results, they remain to depend on the quality and amount of labeled training data, and the typical RF outputs are not geometrically constrained.

Active contour models (ACMs) [16, 17] typically use image edge [18, 19] or region [20, 21] descriptor to drive the active contour toward object boundaries [22, 23]. They have been extensively explored in cardiac segmentation with promising results [24]. For example, Giannakidis et al. [25] employed a user-guided level set based 3D geodesic active contour method to demarcate the left atrial endocardial surface. Avendi et al. [26] and Ngo et al. [27] employed deep learning algorithms combined with deformable models to develop a fully automatic left ventricle (LV) segmentation tool from short-axis cardiac MRI datasets. Yu et al. [28] used sparsity constraints to alleviate gross errors and integrate them seamlessly with deformable models for cardiac motion analysis. Zhou et al. [29] established a new ACM in a variational level set formulation for cardiac CT images segmentation. The limitations of most conventional ACMs include the dependence of a certain contour initialization and the undesirable segmentation results for the images with challenging image conditions, such as intensity inhomogeneity and low tissue contrast.

Atlases may be used within the ACMs or the RFs framework as a priori anatomical information [30]. The segmentation of tissues is performed under the guidance of a single [31] or multiple atlases [32, 33]. With good target-to-atlases registration, atlas-based methods often exhibit good performances for cardiac image segmentation even in the presence of reduced tissue contrast and increased noise [34, 35]. For example, Zhuang and Shen [36] presented a whole-heart segmentation method by employing multimodality atlases based on a multiscale patch strategy and a global atlas ranking scheme. Bai et al. [37] proposed to combine the intensity, gradient, and contextual information into an augmented feature vector and incorporate it into multiatlas segmentation for cardiac MRI. The main drawback of atlases based techniques is the dependence of the segmentation results on the quality of the registration between the target image and the multiple atlases.

To address these limitations, inspired by the pioneering work [26, 38–40], we tackle the complex problem of LA segmentation utilizing a combined RFs and ACM approach. The proposed approach is able to integrate information from multisource images together for a fully automated, accurate, and robust LA segmentation. Specifically, we use the concatenated classification forest to iteratively learn a sequential tissue classifier from each training subject. Inspired by the autocontext scheme [41], the generated tissue probability map at each iteration is further used as additional image source to train the classifier at the next iteration. By fusing the estimations from all trained concatenated classifiers, we can infer the LA structure for a given testing subject. The inferred LA structure is further fed into a volume-scalable ACM as an initial contour as well as a shape prior to accomplish the final segmentation. Compared to the previous methods using RFs and multiatlas for heart chambers segmentation [12, 24], the proposed method allows the effective integration of image information from multiple training subjects without the requirements of registrations. Furthermore, our method reformulates the classification task of RFs as a contour evolution scheme, which is very important for accurate and smooth segmentation of LA images. In addition, in contrast to the previous ACM methods, our method is an automated one and is more robust to low contrast between adjacent tissues. Validations on two public available datasets have demonstrated significant advantages of the proposed method.

The remainder of the manuscript is organized as follows. In Section 2, the proposed method is described in detail. The implementation and results of the proposed method are presented in Section 3, followed by some discussions in Section 4, and finally, Section 5 summarizes this paper.

## 2. Methods

**Figure 1:**
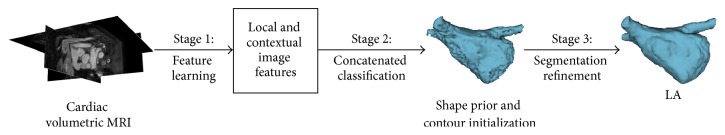
Flowchart of the developed algorithm.

The flowchart of the proposed method is shown in Figure 1. In this paper, we formulate the LA segmentation problem as a hybrid problem with tissue classification and tissue boundary contour evolution. Specifically, with the input of volumetric MRI datasets, the method is carried out in three stages:

(1) A variety of image features are explored from MRI volumes to fully capture both local and contextual image information. These image features are provided as the input to the subsequent stages.

(2) The LA structure is inferred using concatenated random forests (CRFs) in an autocontext scheme [38, 41]. Specifically, the RFs are employed as a concatenated classifier to produce a sequence of tissue probability maps for the LA structure by voxel-wise classification. The LA structure is delineated iteratively by assigning the structure label with the largest probability at each voxel within MRI volumes.

(3) In order to refine the structure labels, the voxel-wise classification is further combined with a contour evolution scheme by feeding the inferred LA structure into a volume-scalable ACM [40]. The final segmentation is accomplished by driving the active contour evolving and converging at the desired position of the LA boundary.

After individual training, the voxel classification, and contour evolution stages of the flowchart offline, the system can be deployed for automatic LA segmentation task. The three stages are further elaborated as follows.

### 2.1. Feature Learning

Denote the vector valued image to be segmented as *I* : *Ω* → *ℜ*^*d*^, where *Ω* ⊂ *ℜ*^3^ is the image domain and *d* ≥ 1 is the dimension of the vector *I*(*x*). In order to fully utilize the information given by the volumetric data, we explore both local and context-aware image features at each voxel, forming a feature map *f* : *Ω* → *ℜ*^*D*_*f*_^. Subsequently, the segmentation is performed in the feature space. Any kind of features from multimodality can be integrated into the proposed framework, such as Fourier [42], wavelet [43], SIFT [44], and HOG [45] features, for tissue segmentation. In this work, we use volume-scalable local robust statistics (VSLRS) [40, 46] to extract the feature vectors due to their insensitivity to image noise and computational efficiency.

Numerically, in computing the robust statistics in local volumes at a controllable scale, and assigning different weights to the data for voxels according to their distance to the central voxel, we define the weighting neighborhood using a nonnegative kernel function* K* such that *K*(*u*) ≤ *K*(*v*) for |*u*| > |*v*| and ∫*K*(*x*)*dx* = 1.

There are various choices for the kernel function. In this work, we use the Gaussian kernel

(1) $$\begin{array}{c} K_{\sigma }\left(\;u\;\right)=\frac{1}{{\left(2\pi \right)}^{n/2}\sigma^{n}}e^{-{\left|u\right|}^{2}/2\sigma^{2}} \end{array}$$

with a scale parameter *σ* > 0.

Then, for each voxel of interest *x* in the image, we define the VSLRS feature vector *f*(*x*) ∈ *ℜ*^*D*_*f*_^ by combining several VSLRS derived in any randomly displaced and randomly scaled local region *R*_random_, or in a scalable local region *R*_centric_ centered at *x*, within a neighborhood *B*(*x*) ⊂ *Ω* around *x*. More explicitly, within the local regions *R*_random_ and *R*_centric_, whose size can be controlled by the scale parameters *σ* of the kernel functions *K*_*σ*_ (1), we first normalize the intensities to have the unit *ℓ*2 norm [47]. Then, we denote

(2) $$\begin{array}{c} VSMEAN\left(\;x\;\right)≔\frac{K_{\sigma }\left(x\right)*I\left(x\right)}{K_{\sigma }\left(x\right)*1} \end{array}$$

as the volume-scalable intensity mean value. In addition, in order to detect the intensity changes yielded by structure changes, meanwhile, eliminating the influence of outliers, the intensity range between the first and the third quartiles, namely, the volume-scalable interquartile range VSIQR(*x*), is calculated as the second feature. Furthermore, the weighted intensity variance is chosen to be the third feature and is calculated as

(3) $$\begin{array}{c} WIV\left(\;x\;\right)≔{\left(\;\frac{K_{\sigma }\left(x\right)*{\left(I\left(x\right)-VSMEAN\left(x\right)\right)}^{2}}{K_{\sigma }\left(x\right)*1}\;\right)}^{1/2}. \end{array}$$

Consequently, we define the VSLRS feature vector *f*(*x*) as

(4) $$\begin{array}{c} f\left(\;x\;\right)=f_{local}\left(\;x\;\right)-bf_{contextual}\left(\;x\;\right),\;b\in \left\{0,1\right\} \end{array}$$

with

**Figure 2:**
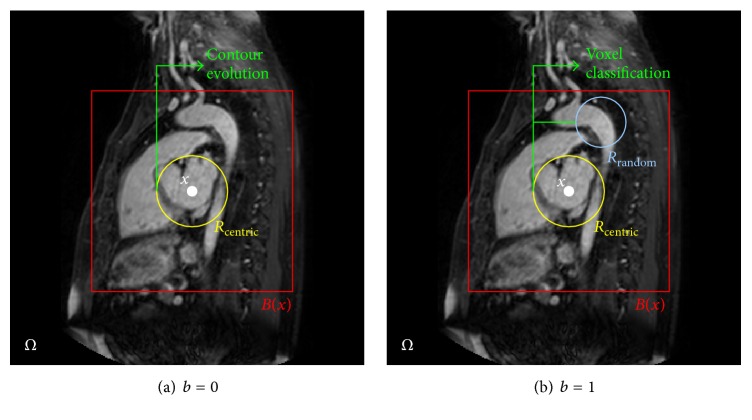
A 2D illustration of the local and contextual features learning scheme. The red rectangle indicated a neighborhood *B*(*x*) around the center voxel *x*. (a) *b* = 0: Local features are captured from a scalable local region *R*_centric_ centered at *x*, within the neighborhood *B*(*x*), and are fed into the contour evolution stage. (b) *b* = 1: Contextual features are captured from randomly displaced and randomly scaled local region *R*_random_, and from *R*_centric_, within the neighborhood *B*(*x*), and are fed into the voxel classification stage.

(5) flocalx=VSMEANx,VSIQRx,WIVxRcentricT∈R3,Rcentric∈Bx,fcontextualx=VSMEANx,VSIQRx,WIVxRrandomT∈R3,Rrandom∈Bx,

where the parameter *b* ∈ {0,1} indicates which contour evolution stage or voxel classification stage the feature vectors are fed into, as shown in Figures 2(a) and 2(b), respectively. Note that the feature vectors derived in *R*_centric_ capture the local image information around a certain voxel, while the feature vectors jointly derived in *R*_random_ and *R*_centric_ capture the contextual image information, which are nonlocal but of short range. In theory, for each voxel in the volumetric data, we can extract an infinite number of such feature vectors by changing the locations and scales of the local regions. In our implementation, we explore the random feature vectors for a voxel from a predefined range with a maximum local region scale of 5 × 5 × 5 and cuboid searching space patch of 31 × 31 × 31 [48].

### 2.2. Concatenated Classification with Autocontext

**Figure 3:**
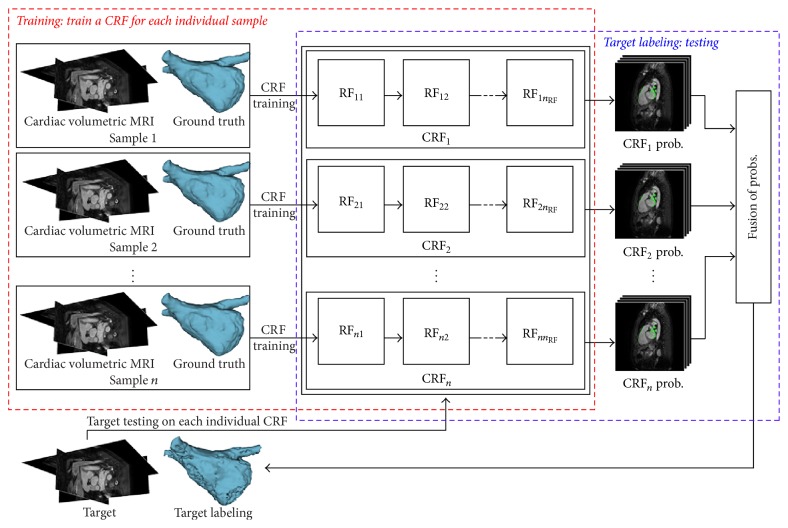
Overview of the proposed concatenated classification framework. A single CRF is trained for an individual sample image. The labeling of a new target is performed by the testing step on each individual CRF and the fusion of the obtained probability maps.

We utilize RFs as a concatenated classifier to infer the LA structure within an autocontext scheme. An overview of the proposed classification framework is illustrated in Figure 3. Similar to the atlas forests [39], we encode a single image by training one corresponding concatenated classifier exclusively on the contextual feature map from the training images. Given a testing image as input, each concatenated classifier returns its own tissue probability map for the target. The target structure is then inferred by fusing the probability maps obtained from different individual concatenated classifiers.

The autocontext metaframework attempts to recursively explore and fuse contextual information, as well as appearance [41]. This means running a sequence of classifiers for each image, such that the probabilistic output of one classifier is fed into the next one for refinement. To this end, we train the concatenated classification forests in the training stage, each with the input of multisource feature maps. For simplicity, we only detail the workflow of one sequence of classifiers. Denote the tissue probability map as *M* : *Ω* → *ℜ*, where *Ω* ⊂ *ℜ*^3^ is the image domain, and let *f*(*I*) and *f*(*M*) be the feature maps for the original image* I* and tissue probability map* M*, respectively. We initiate the process by taking only the appearance feature map *f*(*I*) from the original image as input for voxel-wise classification in the 1st iteration. In the later iterations, the context feature map *f*(*M*) obtained from the previous iteration will act as augmented source feature map. Specifically, the use of these contextual features from tissue probability maps improves the accuracy of the voxel-wise classification by introducing a spatial coherence constraint to those features in addition to providing a better initialization with the tissue probability map from the former classifier. Another consequence of the use of the contextual information is that the registration of the training samples to the target image is avoided while the spatial awareness is preserved.

We employ RFs as a classifier since they can efficiently handle a large number of training data with high data dimension, which is important in the utilization of large numbers of high-dimensional image features [38]. RFs consist of a set of trees, and as a supervised learning technique, they generally operate in two phases: training and testing. In the next section, we will detail our adaption of training and labeling procedures of RFs to the task of LA segmentation.

### 2.3. Random Forests Training and Labeling

During training, each decision tree *t* in the concatenated random forest CRF_*i*_ is trained on the specific* i*-th training sample, which consists of the original volumetric image and the corresponding class label. The original volumetric image is further augmented by tissue probability map as additional source image. Specifically, each decision tree* t* learns a weak class predictor *p*_*t*_(*c*∣*f*(*x*, *I*, *M*)) for a given voxel *x* ∈ *Ω* by using its high-dimensional feature representation *f*(*x*, *I*, *M*).

In the first iteration of training, each tree* t* will learn a class predictor *p*_*t*_(*c*∣*f*(*x*, *I*)) by using only the image appearance feature map from the original image* I*. The training involves recursively splitting the training voxels at each node of the decision tree based on the high-dimensional feature representations of these voxels. In order to improve the generalization, we inject the randomness into the training by using a bagging strategy. Specifically, each tree *t* in CRF_*i*_ has access to a different sample subspace of the specific* i*-th training sample space, and a feature subspace of the whole feature space is randomly selected at each node in the tree. Then, for each sample voxel *x* considered at a given internal node, a binary split is performed independently along each feature of the feature subspace with respect to a certain number of thresholds uniformly distributed along the range of each feature. Along with the split of the sample voxel *x* into its left or right child node, the optimal combination of feature and threshold is learned by maximizing the* information gain* at the node [49]. The tree continues splitting and stops when satisfying certain conditions. Finally, by putting all sample voxels of the* i*-th sample image into the trained forest, we can estimate the tissue probability map *M*_*i*_ from this iteration.

In the later iterations of training, the feature map *f*(*x*, *I*, *M*) explored from both the original image *I* and the tissue probability map *M* which is iteratively updated from the previous iteration is used to learn a class predictor *p*_*t*_(*c*∣*f*(*x*, *I*, *M*)). Then, all later training iterations are performed in the same way as the first iteration.

After training, we can obtain a sequence of classifiers CRF for each training sample and associate each leaf node* l* in the CRF with a class predictor *p*^*l*_*x*_^(*c*∣*f*(*x*, *I*, *M*)) by simply counting the labels of its incoming training samples.

During labeling, each voxel *x* in the target image *I* is labeled by the tree testing on the trained CRFs and the following fusion of the probabilistic estimates from individual concatenated trees. Specifically, by applying the learned split parameters to the high-dimensional feature representation (*f*(*x*, *I*) in the first iteration and *f*(*x*, *I*, *M*) in the later iterations) of a voxel *x*, each tree *t* from a certain CRF yields a class probability *p*_*t*_(*c*∣*f*(*x*, *I*, *M*)).

The probabilistic estimate of the testing voxel *x* from the CRF_*cf*_ with *n*_*t*_ trees at each iteration is then computed as the average of all individual tree predictions, that is,

(6) $$\begin{array}{c} p_{cf}\left(\;c\;|\;f\left(\;x,I,M\;\right)\;\right)=\frac{1}{n_{t}}\overset{n_{t}}{\underset{i=1}{\sum }}p_{t_{i}}\left(\;c\;|\;f\left(\;x,I,M\;\right)\;\right). \end{array}$$

The final probability of the testing voxel *x* is achieved by averaging these probabilities from the *n*_*cf*_ CRFs at the last iteration, that is,

(7) $$\begin{array}{c} p\left(\;c\;|\;f\left(\;x,I,M\;\right)\;\right)=\frac{1}{n_{cf}}\overset{n_{cf}}{\underset{i=1}{\sum }}p_{{CRF}_{i}}\left(\;c\;|\;f\left(\;x,I,M\;\right)\;\right). \end{array}$$

The LA structure is subsequently delineated by selecting arg⁡max_*c*_⁡*p*(*c* | *f*(*x*, *I*, *M*)) for each testing voxel.

### 2.4. Segmentation Refinement: Integrating Contour Evolution into Voxel-Wise Classification

The voxel-wise classification is performed for each voxel independently, which might introduce artificial anatomical errors in the delineated LA structure [38, 50]. To address this limitation, we employ a volume-scalable ACM combined with the LA structure inferred from the previous stage for segmentation refinement in the final stage. ACMs can drive the active contour evolving and converging at the desired position of the object boundary by minimizing an energy functional. Compared with RFs, ACMs can provide geometrically constrained segmentation results with subpixel accuracy. Most conventional ACMs are not fully automated due to the necessity for the contour initialization and usually fail to segment cardiac MRI with intensity inhomogeneity and low tissue contrast. We solve these issues by using the inferred LA structure as an initial contour, and also as a shape prior integrated into a volume-scalable energy functional.

Denote the target object volume and the background volume as *Ω*_1_ ⊂ *Ω* and *Ω*_2_ ⊂ *Ω*, respectively. In particular, *Ω*_Inf_1_ and *Ω*_Inf_2_ indicated the seeded LA volume and the seeded background volume, respectively, which are inferred from previous stage. Then, with the VSLRS feature vector defined in (4), each voxel *x* can be characterized by combining the probability distribution functions (PDFs) of the feature vectors derived in the inferred volumes with that derived in a neighborhood around voxel *x*. The characterization of voxel *x* is then described as follows:

(8) Pix=1−ω1ΩInf_i∑z∈ΩInf_ipfx−fz+ω∫ΩiKηx−ypμix−fydy,i=1,2,

where in the first term *z* is the seed voxel that belongs to inferred volumes and the second term is a weighted average of the probability distribution *p* in a neighborhood of voxel *x*, whose size is controlled by the scale parameter *η* of the kernel function given by (1). Moreover, *μ*_*i*_ in the probability density approximates image characters in local volume *Ω*_*i*_. Finally, *ω* is a positive constant (0 ≤ *ω* ≤ 1) which balances the importance of inferred structures and the local volumes [40].

Let *ϕ* be the level set function and *H*(·) be the Heaviside function. In particular, denote the reference level set function corresponding to the LA structure inferred from the previous concatenated classification stage as *ϕ*_ref_. The energy functional *F*(*ϕ*, *P*_1_, *P*_2_) can be expressed as

(9) Fϕ,P1,P2=Eϕ,P1,P2+α1Lϕ+α2Pϕ+α3Sϕ,

which is a combination of the volume-scalable fitting energy functional

(10) Eϕ,P1,P2=−λ1∫∫Kηx−yP1y·Hϕydydx−λ2∫∫Kηx−y·P2y1−Hϕydydx,

smoothness term

(11) $$\begin{array}{c} L\left(\;\phi \;\right)=\int \left|\;\nabla H\left(\;\phi \left(\;x\;\right)\;\right)\;\right|dx, \end{array}$$

level set regularization term

(12) $$\begin{array}{c} P\left(\;\phi \;\right)=\int \frac{1}{2}{\left(\;\left|\;\nabla \phi \left(\;x\;\right)\;\right|-1\;\right)}^{2}dx, \end{array}$$

and a priori shape energy term

(13) $$\begin{array}{c} S\left(\;\phi \;\right)=\int -\ln\left(\;p\left(\;\phi \left(\;x\;\right)-\phi_{ref}\left(\;x\;\right)\;\right)\;\right)dx. \end{array}$$

Here, *α*_*i*_'s, *i* = 1,…, 3, and *λ*_*j*_'s, *j* = 1,2, are positive constants; *P*_1_ and *P*_2_ are two values defined in (8) that characterize image voxels with inferred volumes and neighbor volumes. Also *K*_*η*_ and *p* are the kernel functions given by (1), which control the size of a local volume centered at the voxel *x* and estimate the level set shape similarity, respectively. Then, by minimizing the energy functional *F*(*ϕ*, *P*_1_, *P*_2_), we can obtain the entire object boundary.

We solve the energy functional minimization problem by using the standard gradient descent method. Keeping *ϕ* fixed and minimizing the energy functional *F*(*ϕ*, *P*_1_, *P*_2_) in (9) with respect to the functions *P*_1_ and *P*_2_, we deduce the following optimal expressions for the functions *P*_1_ and *P*_2_ that minimize *F*(*ϕ*, *P*_1_, *P*_2_):

(14) P1x=1−ω1ΩInf_1∑z∈ΩInf_1pfx−fz+ω∫Kηx−ypμ1x−fy·Hϕydy,P2x=1−ω1ΩInf_2∑z∈ΩInf_2pfx−fz+ω∫Kηx−ypμ2x−fy·1−Hϕydy,

with

(15) μ1x=∫Kηx−yfyHϕxdy∫Kηx−yHϕxdy,μ2x=∫Kηx−yfy1−Hϕxdy∫Kηx−y1−Hϕxdy.

Keeping *P*_1_ and *P*_2_ fixed, we minimize the energy functional *F*(*ϕ*, *P*_1_, *P*_2_) in (9) with respect to *ϕ* using first variation of *F* by solving the gradient descent flow of *ϕ* as follows:

(16) ∂ϕ∂t=δϕλ1e1−λ2e2+α1δϕdiv⁡∇ϕ∇ϕ+α2∇2ϕ−div⁡∇ϕ∇ϕ−α3ϕ−ϕref,

where *δ* is the Dirac delta function and *e*_1_ and *e*_2_ are the functions

(17) $$\begin{array}{c} e_{i}\left(\;x\;\right)=\int K_{\eta }\left(\;x-y\;\right)P_{i}\left(\;y\;\right)dy,\;i=1,2 \end{array}$$

in which *P*_1_ and *P*_2_ are given by (14).

In the last stage of the proposed workflow, the final refined segmentation result is achieved by evolving the level set equation (16) with initialization of level set function *ϕ* obtained from the inferred LA structure.

## 3. Implementation and Experimental Results

### 3.1. Implementation Details

We evaluate our approach on the cardiac volumetric MRI datasets from the STACOM 2013 LA Segmentation Challenge [7] and the HVSMR 2016 Whole-Heart and Great Vessel Segmentation Challenge [51], respectively. Specifically, we artificially enlarge the training dataset from the STACOM 2013 Challenge by a factor of ten using image processing techniques such as translation, changing the resolution by downsampling or upsampling, and changing the voxel intensities based on the standard principal component analysis (PCA) [52]. Then, we use the augmented training dataset for algorithm training and parameters tuning. The subsequent validations are performed on the testing dataset from the STACOM 2013 database and the training and testing datasets from the HVSMR 2016 database, respectively. The corresponding labels released with the STACOM 2013 Challenge and the expert manual segmentation of the LA structure for each case in the HVSMR 2016 database are provided and considered as the ground truth.

In our implementation, we train *n*_*cf*_ = 10 CRFs, each of which corresponds to one of the training subjects of the enlarged training set. We use *n*_*t*_ = 5 classification trees for each CRF at each iteration and set the iteration number as *n*_iterations_ = 5. The maximum depth of classification tree and the minimum number of samples contained in leaf node are restricted to 50 and 8 [39], respectively. For each classification tree, we randomly sample all the corresponding categorical training voxels with replacement for the target and background class labels, respectively, from each training subject. During the tree training, each node considers *n*_*f*_ = 10,000 randomly sampled VSLRS features with their respective *n*_thresholds_ = 20 randomly distributed thresholds to determine the optimal split functions. The parameters in the volume-scalable ACM are the tweaked parameters determined empirically [40] during training as

(18) σ=0.5,η=3.0,λ1=0.2,λ2=1.0,Δt=0.1,ω=0.4,α1=α2=α3=1.0,

for the best segmentation results. The influence of the parameters in our combined approach on the segmentation results will be discussed in “Discussion.”.

In addition, two metrics, surface-to-surface distance (S2S) [7] and Dice coefficient (DC), with respect to the ground truth are computed for quantitative evaluation of the proposed method. The volume rendering is implemented using the “Model Maker” module in 3D Slicer [53].

### 3.2. Illustrative Results

**Figure 4:**
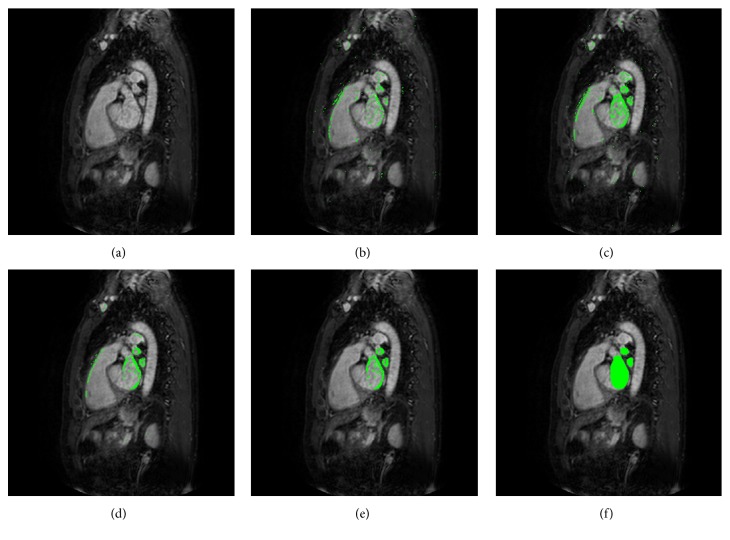
The tissue probability maps estimated from individual CRF and multiconcatenated RFs for a target subject. (a) The axial slice from the original volumetric data. (b)–(d) Voxels with more than 0.6 confidence (green spots) in the outcomes of an individual CRF with iterations 1, 2, and 5, respectively. (e) Fusion of each individual CRF. (f) Ground truth.

Figure 4 illustrates the impact of individual CRF and fusion of multiple CRFs on the segmentation results for a testing subject. The original target image and corresponding ground truth are shown in Figures 4(a) and 4(f), respectively. The tissue probability maps estimated from each iteration of an individual CRF are shown in Figures 4(b)–4(d).  Figure 4(e) shows the tissue probability map estimated from fusing the final iterations of each individual CRF in the multiconcatenated RFs. For clarity, we only highlight the voxels of which the confidence is higher than 0.6 in the probability maps with green spots.

**Figure 5:**
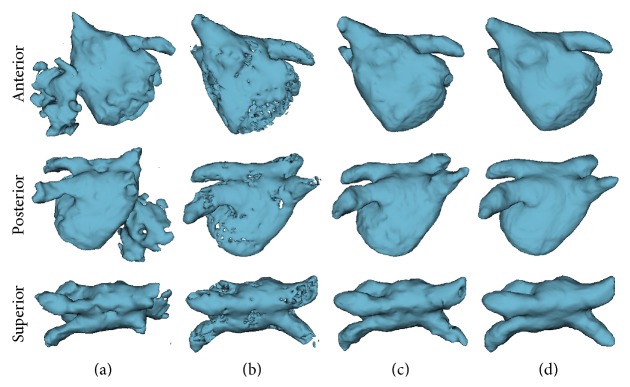
Visual comparison of the proposed method with different components integrated on case B003 of the STACOM database. (a) Outcome of the volume-scalable ACM without shape constraint; (b) result of the CRFs without contour refinement (stage 2); (c) the combined CRFs and volume-scalable ACM (final stage); (d) ground truth.

In order to better understand the influence of the different stages of the proposed method, Figure 5 shows the outcomes of the volume-scalable ACM without shape constraint, the CRFs without contour refinement (stage 2), and the integrated CRFs and volume-scalable ACM (final stage) on case B003 of the STACOM database, respectively. The anterior, posterior, and superior views of the 3D segmentation results and the ground truth are provided in the 1–3 rows, respectively.

**Figure 6:**
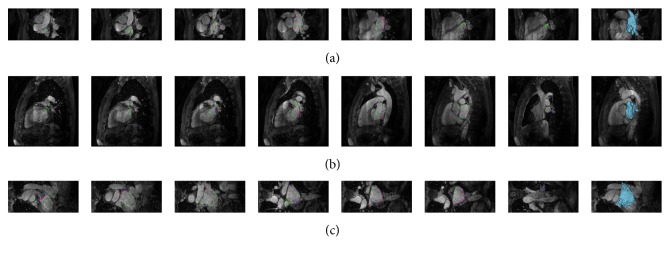
Multiple slices of the 3D segmentation results for case B006 of the STACOM database. The final contours (shown as colored contours in the 1–7 columns) and the 3D surface models (shown as blue volumes in the last column) overlaid on the corresponding axial slices (a), the sagittal slices (b), and the coronal slices (c), respectively. The red contours are generated by the proposed algorithm, while the blue contours represent the ground truth, and the green contours are where the algorithm ones coincide with the ground truth.

To demonstrate the advantage of the proposed method in terms of segmentation accuracy and robustness more clearly, we show multiple slices of a low quality MRI dataset in three standard views and the final segmentation result obtained by the proposed method (compared with the ground truth segmentation) in Figure 6.

### 3.3. Quantitative Results

**Figure 7:**
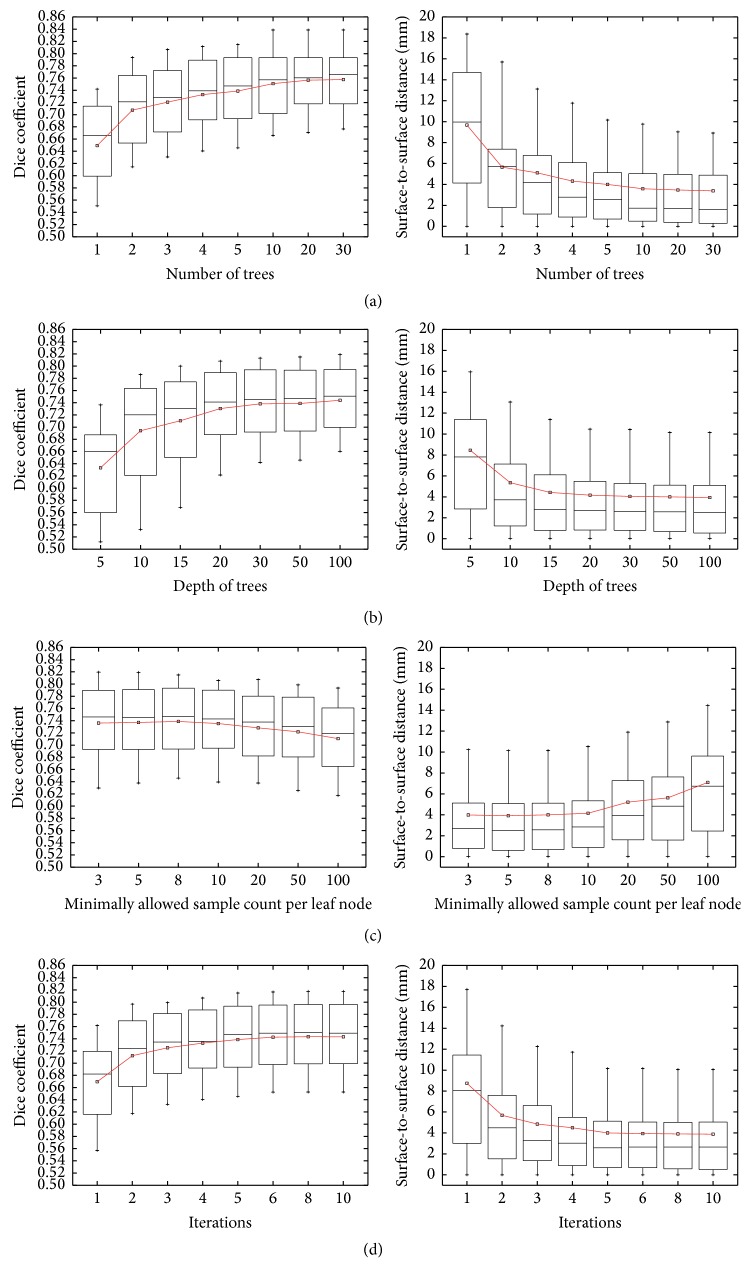
Impact of 4 different parameters in the concatenated classification stage: the number of trees used per RF (a), the maximally allowed depth for each tree (b), the minimally allowed sample count per leaf node (c), and the iteration number of the concatenated scheme (d).

Figure 7 illustrates the influence of different parameters (e.g., the number of trees, depth of trees, minimally allowed sample count, and the iteration number of the concatenated scheme) on the segmentation results of the proposed CRFs. Values for these parameters were determined via leave-one-out cross-validation on the artificially augmented training dataset of the STACOM database, according to the parameter tuning method described in [54]. In this experiment, only the trends of the influence are interested. Therefore, during parameter tuning, when a certain parameter was tuning, the other parameters were set to their respective fixed values instead of the optimal values for the best segmentation results. Please note that we only test the parameters of the CRFs here, while the parameters of the volume-scalable ACM in the last stage of the proposed scheme were discussed in our former paper [40].

In addition, the proposed method was compared to the standard ACM-based method [55, 56], RF-based method [11, 13], and multiatlas-based method [30, 32], respectively. We quantitatively evaluate the performance of different methods by employing the Dice coefficients (DC) and surface-to-surface distance (S2S), as shown in Table 1. The Proposed 1 and Proposed 2 represent the proposed method* without* (stage 2) and* with* (final stage) contour refinement, respectively, as described in “Segmentation Refinement: Integrating Contour Evolution into Voxel-Wise Classification.”

In the current study, the algorithm running time of the proposed method was recorded from our experiments with c++ code run on a computer cluster, with 4.2 GHz Inter Core i7 processor and 32 GB RAM, with Visual Studio 2015 on Windows 7. The average training time for one forest in the second stage is around 1.5 h. For each of *n*_iterations_ = 5 iterations, we trained *n*_*cf*_ = 10 CRFs. By parallel training all these forests, the elapsed time of each iteration is around 1.5 h, resulting in total training times of approximately 7.5 h. Once trained, the approximated elapsed times of the LA segmentation in a typical cardiac volumetric MRI are as follows: LA structure inferring (CRF labeling): 3 min, segmentation refinement (volume-scalable ACM): 30 s.

## 4. Discussion

We have presented a combined approach to effectively integrate voxel-wise classification and contour evolution for LA volumetric MRI segmentation. Specifically, we employ a RF technique to effectively infer LA structure. Due to several challenges including the intrinsic limitation of the classification scheme and the limited number of training data, the inferred shape is not satisfied enough. Thus, we refine the delineated shape by using ACM to bring more accuracy to the segmentation result.

### 4.1. Impact of the Concatenated RFs

As seen in Figures 4(a)–4(d) and 4(f), the accuracy and sharpness of the inferred tissue probability maps are gradually improved along with the iterations of individual forest prediction. Also, fusion of individual forest predictions further improves the inferred tissue structure, as can be seen in Figures 4(e) and 4(f). An explanation for these observations is that, in the first iteration of individual CRFs, only the image appearance features are used to generate the tissue probability map which results in many false positive results along the edges in Figure 4(b). In the later iterations, the concatenated scheme refines the inferred structure (tissue probability map) by recursively integrating the tissue probability maps estimated from the previous iteration and the appearance feature map extracted from the original image. As we can see from Figures 4(c) and 4(d), the tissue probability maps of one-individual CRFs are gradually improved with iterations (*n*_iterations_ = 5 in current implementation). At the end of the concatenated classification procedure, each individual's CRFs (*n*_*cf*_ = 10 in current implementation) will generate a different soft segmentation in a tissue probability map fashion. In the subsequent fusion procedure, multiple individual tissue probability maps are averaged by (7) where some false-high probabilities of the testing voxels from one probability map may be corrected by the other probability maps. As we can see from Figure 4(e), the averaged tissue probability map becomes more accurate, by comparing with the ground truth shown in Figure 4(f).

### 4.2. Influence of the CRFs and the ACM

As seen in Figure 5(a), although the initial seeds and the iteration number of the volume-scalable ACM have been determined sophisticatedly, the leakage of the segmentation result is severe due to the similar intensities of the blood pools. Furthermore, as a consequence of insufficient contour evolution, the image is undersegmented which appears as the missing of parts of the PV. Alternatively, benefiting from the context-aware image information and the concatenated training and testing schemes, the CRFs do provide certain discriminative advantage in terms of controlling leakage. However, because the classification scheme of the RFs labels each individual voxel exclusively, the outcome of the concatenated classification scheme is not geometrically constrained (see Figure 5(b)). Finally, as seen in Figure 5(c), the combined CRFs and volume-scalable ACM brought more accurate geometrically constrained segmentation result. This is due to the fact that the a priori shape constraint term in (13) significantly prevents the active contour from leaking to the ventricle region; meanwhile, the ACM fills the holes of the CRFs result and refines the details of the result.

### 4.3. Segmentation Accuracy and Robustness

It can be seen from Figure 6 that; despite the great challenge of these image slices due to their lower spatial resolution and intensity inhomogeneities, the corresponding segmentation results are quite consistent with the ground truth. The proposed method successfully recovers the smooth boundary of the LA in the volumetric MR image.

### 4.4. Impact of the Parameter Settings

In Figure 7, we show the impact of different parameter settings on the accuracy. We first study the impact of the number of trees per RF on segmentation accuracy in Figure 7(a). We find that there is a clear increase in accuracy from using 1 tree (0.6493 ± 0.0673) to using 2 trees (0.7074 ± 0.0614), measured by the average DC value, and the accuracy is improved as the number of trees increased. In addition, we see that the performance is quickly getting stable beyond a certain number of trees. This effect is probably due to the strategy of fusing multiple forests. In this paper, we conservatively choose 5 trees for each forest in each iteration. Next, we study the effect of the maximally allowed depth for each tree (b). We find that the performance is gradually improved from depth of 5 to depth of 30. In order to make the depth of the trees accommodate for the amount of the training samples as well as the minimal sample count per leaf, we set this parameter to 50 in this paper. Further, we test the impact of the minimally allowed sample count per leaf node (c). It can be seen that the performance is improved by decreasing the minimal sample count down to 8, while a small setting of this parameter such as 3 will be likely to be overfitting. Finally, we analyze the influence of the iteration number on the segmentation accuracy. In Figure 7(d), we can see that the more the iterations, the better, and the performance becomes stable after a certain number of iterations. In particular, we see significant improvement from the 1st iteration to the 2nd iteration. This effect is due to the use of the previously estimated tissue probability maps for subsequent voxel classification. Their results further demonstrate the effectiveness of the concatenated classification scheme for segmentation.

### 4.5. Method Comparison

Computed metrics in Table 1 show that, even for the proposed method without final contour refinement (the last second row), it produces a competitive accuracy on the validation databases with an overall accuracy of 0.7205 (in terms of DC) and 4.405 mm (in terms of S2S), respectively. Although the DC values by the Proposed 1 method is not satisfied enough, it can be improved by the subsequent contour refinement procedure. As demonstrated in Table 1, the DC values by the proposed CRFs are low which are mainly caused by the hollow part in the segmented LA body as seen in Figures 4 and 5. However, the delineated outer contour of the LA structure shows great agreement with the ground truth which also can be seen in Figures 4 and 5 and further demonstrated by the S2S values shown in Table 1. Therefore, the inferred shape can provide good initial contours to the subsequent contour evolution. Consequently, the integrated volume-scalable ACM (the last row) in the Proposed 2 method achieves a superior accuracy with an overall DC of 0.9227 and S2S of 1.14 mm, as other standard methods cannot fully utilize the contextual volumetric image information for guiding the segmentation.

Though it may not be reliable for comparing different methods by utilizing different datasets, the performance of the proposed method seems to be competitive with some of the latest results [24, 57]. For example, one state-of-the-art method [36] reports a result of 0.878 ± 0.0624 and 1.34 ± 1.28 mm for the DC and S2S measures, respectively. The proposed method outperforms the referenced method in terms of mean and standard deviation values, due to the concatenated classification and contour evolution scheme, and the usage of context-rich information for interpreting the LA structure.

### 4.6. Computational Time

The main computational cost in the proposed method is for training the CRFs. Although the training procedure of the proposed method can be implemented offline within a reasonable time, their efficiency is never perfect. Nevertheless, the computational efficiency of the training stage can be improved by using GPUs in the near future. In testing, the algorithm running time to perform LA segmentation in a typical cardiac volumetric MRI can be within 4 min, which was mostly taken by the CRFs labeling and volume-scalable ACM evolving. Note that the integrated ACM in the final stage of the proposed method can converge in less iteration than pure ACM, due to the imposed initial contour and shape prior which were inferred from previous stage. Inspired by the recent LightGBM [58] and sparse representation techniques [59], we will further optimize the proposed work.

Finally, one of the difficulties in developing RFs based cardiac MRI segmentation techniques is the need for a number of data for training and validation. Many iterations of training on limited training samples may lead to overtraining. Although the bagging strategy of the RFs can guarantee the diversity of trees by randomly selecting a subset of samples for the training of each tree, overfitting is still an important issue for the proposed concatenated scheme. To cope with this limitation, we artificially enlarge the training dataset by a factor of ten using image processing techniques such as translation, changing the resolution by downsampling or upsampling, and changing the voxel intensities based on the standard principal component analysis and feed the CRFs with the augmented sample dataset during training as described in “Implementation Details.” However, the overfitting issue still exists in our current implementation, and, accordingly, we observe a higher accuracy of the proposed method on the STACOM 2013 dataset which provides the training samples than the accuracy on the HVSMER 2016 dataset which only provides the testing images as shown in Table 1. A possible remedy would be to make each CRF access more data during training.

## 5. Conclusion

In summary, in this work, we propose to fully automatically segment the LA structure from cardiac volumetric MRI through a novel combined RFs and ACM approach. In contrast to previous RFs based methods that define the segmentation problem as a classification task, our approach refines the voxel-wise classification through a contour evolution scheme and therefore achieves geometrically constrained segmentation results. Also, while current standard learning schemes pool training set indiscriminately across all training subjects and encode image points statically, our approach has a flexible training samples selection scheme and explores flexible representations of the individual points in the multisource images from the contextual image information. Compared to standard ACMs, which rely on certain contour initialization and usually fail to segment cardiac MRI with challenging image conditions, our approach has a number of advantages, such as the ability to automate the contour initialization process, and brings more accuracy and robustness through sophisticated feature learning and shape prior integrating schemes.

As demonstrated in our experiments, the proposed method is able to segment the LA volumetric MR images with challenging image conditions and has desirable performance in terms of accuracy and robustness. The proposed method achieved a high accuracy of 0.9227 ± 0.0598 and 1.14 ± 1.205 mm for the LA segmentation, measured by DC and S2S values, respectively. Comparative experiments have demonstrated the advantages of the proposed method over other state-of-the-art automated segmentation methods. The scalability of our method on a larger scale of multimodality clinical images will be investigated in our future work.

## Figures and Tables

**Table 1:** Dice coefficients (DC) and surface-to-surface distance (S2S) of different methods on the STACOM and HVSMR datasets.

Method	STACOM 2013		HVSMR 2016		Total	
	DC	S2S (mm)	DC	S2S (mm)	DC	S2S (mm)
ACM	0.7132 ± 0.2017	8.95 ± 7.64	0.728 ± 0.2102	8.49 ± 7.13	0.7206 ± 0.206	8.72 ± 7.39
RF	0.6327 ± 0.1372	6.42 ± 5.37	0.6117 ± 0.1521	6.92 ± 5.17	0.6222 ± 0.1447	6.67 ± 5.27
Multiatlas	0.693 ± 0.2364	5.82 ± 4.95	0.6792 ± 0.253	5.73 ± 5.11	0.6861 ± 0.2447	5.775 ± 5.03
Proposed 1	0.7374 ± 0.0712	4.15 ± 2.87	0.7036 ± 0.072	4.66 ± 2.95	0.7205 ± 0.0716	4.405 ± 2.91
Proposed 2	0.9342 ± 0.0521	1.01 ± 1.17	0.9112 ± 0.0675	1.27 ± 1.24	0.9227 ± 0.0598	1.14 ± 1.205
